# Macro-Demographics and Ageing in Emerging Asia: the Case of Indonesia

**DOI:** 10.1007/s12062-022-09358-6

**Published:** 2022-04-04

**Authors:** George Kudrna, Trang Le, John Piggott

**Affiliations:** CEPAR, University of New South Wales (UNSW), Sydney, Australia

**Keywords:** Population ageing, Asia, Indonesia, Household survey data

## Abstract

In common with a number of other emerging economies in South East Asia, Indonesia is confronting rapid demographic transition at a low level of per capita income. The fourth largest country in the world by population size, Indonesia will face new challenges for fiscal sustainability and policy design, as in coming decades its labour force begins to shrink, and the older population becomes relatively more numerous. In this paper, we demonstrate how strong data sources, from international agencies, national sources, and surveys of the Health and Retirement Study (HRS) family, are available and can be combined to generate a statistical profile of an emerging economy. Such profiles have value in themselves but can also be used as the basis for specifying macroeconomic models of demographic transition, of the overlapping generations (OLG) type, and for various other purposes. The profile presented here will serve to inform both policymakers and the broader community of the long-run trends which will inexorably impact Indonesian society in coming decades. It indicates that major social protection policy development will be needed over the next period to avert widespread hardship, especially among older cohorts.

## Introduction

The Asian region is experiencing rapid population ageing, with the number of people in their 60s and older expected to more than double, from about 520 million today to about 1.2 billion by 2050 (United Nations (UN) 2019). For many populous Southeast Asian economies, this demographic transformation is occurring simultaneously with major shifts in labour markets, technology and formalisation, and against a backdrop of deficient social protection policies that do not cover a large proportion of older adults (World Bank, 2016). Developing or reorienting policy to anticipate and mitigate the social and economic impacts of ageing will be crucial.

This paper documents the economic and social circumstances prevailing in Indonesia, a low-income country with a population of 273 million, in the context of rapid demographic transition. In common with a number of other emerging economies in East and South East Asia, most older adults in Indonesia are experiencing significant hardship, with nearly half either in poverty or vulnerable to poverty. Economic growth per se does not seem thus far to have led to an improvement in the circumstances of these cohorts, and ongoing societal ageing suggests that this situation will become more critical in the next couple of decades.

But it is important to recognise that gathering information on older cohorts alone does not provide an adequate account of the underlying socioeconomic dynamics. At the very least, information about younger cohorts – changes in educational levels, the development of a formal sector workforce, and so on – is a critical input into comprehending the potential for public sector intervention to address the circumstances of Indonesia’s older people, both now and into the future.

By way of introduction, it is instructive to report levels and trends in inequality. While Indonesia’s official poverty rate was 9.2% in 2019, around 20% of the population remains vulnerable to becoming poor, measured by the vulnerability line equal to 1.5 times the national poverty line (World Bank, 2020a). Official poverty rates among the elderly are reportedly much higher, with about 42% of the elderly aged 60 years and above are either in poverty or living just above the poverty line (Priebe & Howell, 2014), pointing to the need for more comprehensive social protection for the elderly.^1^

Increasing inequality is also a feature of Indonesia’s recent history. According to Organisation for Economic Co-operation and Development (OECD) (2019), the Gini coefficient increased from 0.3 in 2004 to 0.41 in 2014, one of the fastest increases in the Southeast Asian region. This scenario is sometimes associated with a “middle income trap”, in which rising inequality retards economic development.

**Fig. 1:**
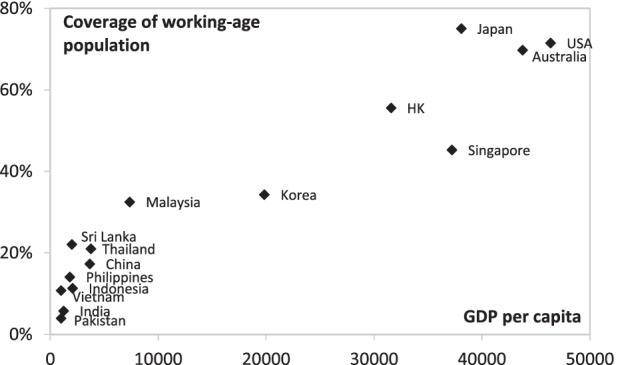
Pension coverage of working-age population (mid-2000s). *Source*: Chomik and Piggott (2015)

In part, these figures reflect a very undeveloped social protection system, especially with regard to those elements of social protection directed to the elderly. Many emerging economies in the region share this characteristic. Chomik and Piggott (2015) provide some detail to give this proposition substance. To take just one example, Fig. 1 depicts the social security coverage of the population by per capita income for a range of Asian countries. The large group of countries clustered in the bottom left-hand corner of the chart provides evidence of low coverage rates.^2^

Sustainable social policy development, however, requires a strong evidence base around prevailing long term economic and demographic forces, and an economic framework within which to test policy proposals. In common with other countries in the region, Indonesia is fortunate in having a longstanding and comprehensive survey which is more or less nationally representative, the Indonesian Family Life Survey (IFLS) (Strauss et al., 2016). Also important are the United Nations demographic data and projections (UN, 2019). Along with other national surveys, these provide data which allow the development of an economic and demographic profile within which to embed social policy proposals.

Constructing an informed macro-demographic profile of an emerging economy can be a daunting challenge, but is an essential pre-cursor to evidence based social policy development. This is the task of the present paper. This work was initially motivated to provide context and data for a major research effort around a detailed macroeconomic model designed to identify the most appropriate social protection structures for Indonesia. But it has generated insights into the circumstances of older cohorts, and associated changes through time, which have value in their own right.

The demographic and household survey data sources identified above is sufficient to craft a profile of older cohorts in Indonesia, within a life-course context of changing education and labour force participation. We focus on demographic change, labour force and older adults, who, for the purposes of this paper, can be thought of as those aged 50 and above. In this sense, the paper complements the account of India and its longitudinal survey, LASI provided by Bloom et al. (2021). We find that:

- Indonesia will undergo pronounced population ageing driven by a reduction in total fertility rate. For example, the aged dependency ratio (65+/15–64) is projected to increase from less than 10% (in 2020) to over 46% in 2100. This is also attributed to an increasing life expectancy, particularly at older ages. For those at age 65, life expectancy is projected to increase by almost 20 years in 2100 (which is almost double the expected lifespan in the middle of the twentieth century). Indonesia’s total population has also quadrupled to 273 million (in 2020) since 1950 and is projected to increase to 320 million in 2100. However, the annual population growth rate will become negative, reaching −0.3% in 2100 due to population ageing.
- Importantly, drawing on IFLS household survey data, this demographic transition is occurring in an economy where the large majority of the labour force operates in informal employment, not covered by a formal retirement income policy or, currently, a social pension.
- At older ages, people continue to derive their income mainly from employment, along with private transfers from their adult children and these two income sources will be impacted by fewer adult children (to provide private transfers) and longer lifespans (affecting the labour supply of older adults).These findings emphasize a pressing need for major and sustainable social policy development over the next two decades to mitigate negative social and economic implications of this demographic shift and to avert large-scale poverty among older cohorts.

The paper is structured as follows. In the next section, data sources for Indonesia are introduced: the UN demographic data base, the IFLS, and the National Labour Force and Socio-Economic Surveys are all discussed. Section 3 documents the demographic change, presenting the past, current and future demographic developments in Indonesia, and provides insights into the Indonesian labour force, reporting on formal-informal labour, skills and earnings. Section 4 focuses on older adults in Indonesia, documenting their characteristics, employment and income sources. Both Sections 3 and 4 use the IFLS. The final section provides interim conclusions, including a summary of the key stylized facts derived from the Indonesian population, its labour force and older adults.

It should be noted that much of the statistical analysis for this research was carried out prior to the onset of COVID-19, and that our estimates do not take account of the impact of the pandemic on mortality or fertility. In the context of the long runs that are the focus of the paper, this may not be too serious a limitation, but it should be borne in mind.

## Demographic and Household Survey Data

This section describes the following data sources: The United Nations World Population Prospects data (UN, 2019) and the Indonesia Family Life Survey (IFLS). The UN data are used to document the demographic change in Indonesia and other emerging Asian economies; the IFLS allows analysis of the Indonesian labour force and older adults. We conclude this section by also outlining the main nationally representative household surveys in Indonesia – the National Labour Force Survey (SAKERNAS) and the National Socio-Economic Survey (SUSENAS).

### UN World Population Prospects

The 2019 Revision of World Population Prospects is the twenty-sixth round of official United Nations population estimates and projections for the world population, different regions and individual countries around the world.^3^ It provides population estimates from 1950 to 2020 and projections up to 2100. Projections allow for several different variants, including different fertility rate assumptions.

In Section 3, we use UN data for Indonesia and other emerging Asian countries. In relation to demographic inputs, we focus on (the changes in) fertility and survival rates. For the demographic outcomes, we report on (the changes in) the total population as well as cohort shares from 1950 to 2020 and to 2100. We also provide key results for alternative projections, emphasizing the importance of past fertility trends and future fertility assumptions.

The UN projections are highly useful constructs, especially for comparative analysis. But they should not be regarded as firm predictions. They make many assumptions, including about the fertility behaviours of women who have not yet been born, and take no account of national or regional developments that may impact fertility or mortality.

### Indonesian Family Life Survey

The Indonesian Family Life Survey (IFLS) is an ongoing longitudinal survey in Indonesia, representative of about 83% of the Indonesian population and containing over 30,000 individuals living in 13 of the 27 provinces in the country (documented by Strauss et al., 2016).^4^ It consists of five waves that were initiated in 1993, 1997, 2000, 2007, and 2014.

These surveys are rich-information socio-economic surveys which collect a wide range of data for studying life cycle behaviour and outcomes for the Indonesian population.^5^ Data on employment, labour force participation, education, health, income, expenditure, housing, fixed assets and durable goods are reported. Coverage of poverty alleviation programs, general economic conditions, agricultural production, local infrastructure and transportation are also reported, and in combination these allow us to construct a comprehensive panel data set.

IFLS is extensively used in sections 3 and 4, focusing on IFLS survey waves 3 to 5 for years 2000, 2007 and 2014.^6^ These surveys and their procedures were reviewed and conducted by RAND corporation, and in Indonesia at the University of Gadjah Mada. Details on the variables used from this data source are provided in Section 3 (for the labour force) and Section 4 (for older adults). Below, we provide the definition of informal labour used in our empirical investigation with the IFLS.^7^

#### Definition of Informal Employment

An important set of data relates to the informal labour force in Indonesia, because informality is generally associated with low productivity. Analysis requires a definition of informal employment. According to the international statistical definition of informal employment by International Labour Organization (ILO, 2002, p.124), “employees are considered to have informal jobs if their employment relationship is not subject to national labour legislation, income taxation, social protection or entitlement to certain employment benefits”.^8^ Due to the wide diversity of social and economic situations in different countries, this broad guideline leaves the operational and specific criteria for establishing informality for individual countries, with specific definitions depending also on the actual data availability.

For Indonesia, informal workers are defined based on the legal status of enterprises, that is, whether enterprises are registered (UU Ketenagakerjaan No. 13 of 2003 by the Ministry of Manpower and Transmigration, discussed by Nasip & Pradipto, 2016). Furthermore, since 1993, the government has been requiring registered employers to provide health benefits to employees through the Employees’ Social Security System. Under that regulation, formal workers are entitled to receive health benefits provided by their employers.

In this paper, we use medical benefits from employers in the form of health insurance and/or any other medical expenditure as an indicator of formality. If a person reports that they have received either health insurance or medical expenditure from their employer, we code them as a formal worker.^9^ Given the availability of relevant information in IFLS, this method of identifying informal workers is in line with the general method implemented by ILO (2018b), which utilizes the “entitlement to and benefit from paid sick leave” as an indicator to determine informal employment for employee.

However, if a person reports that they are self-employed, they will not be asked about medical benefits from employers. For those people, we code them as informal workers, given the fact that most of them are working in agriculture as farmers or small and unregistered household businesses. The same assumption has been used by McKee (2006) and Cuevas et al. (2009) to determine the informality for self-employment in Indonesia, where more than 99% of jobs in agriculture are informal.^10^

In our follow-up (economic model-based) studies, we use this definition of informal employment as a proxy for those operating outside of the retirement income policy in Indonesia. Capturing those operating in the informal sector, and also those in the formal sector but with no pension policy coverage, is important for our follow-up, quantitative analysis of ageing and retirement income policy in Indonesia.^11^

### National Labour Force and Socioeconomic Surveys

The two main nationally representative household surveys in Indonesia are the National Labour Force Survey (SAKERNAS) and the National Socio-Economic Survey (SUSENAS). SAKERNAS was initiated in 1976 to cover national labour market characteristics of all working age individuals within sampled households.^12^ It has generally been conducted on an annual basis since 1976 and on a biannually basis since 2005 by Statistics Indonesia (BPS, Badan Pusat Statistik), drawing on either quarterly or annual observations. It covers around 220,000 individuals and 70,000 households across the nation.

Another BPS survey, SUSENAS also provides information on the labour market.^13^ SUSENAS is a series of large-scale multi-purpose socioeconomic surveys initiated in 1963–1964 and fielded every year or two since then. Since 1993, SUSENAS surveys cover a nationally representative sample typically composed of 200,000 households. Each survey contains a core questionnaire which consists of a household roster listing the sex, age, marital status, and educational attainment of all household members, supplemented by modules covering about 60,000 households that are rotated over time to collection additional information such as health care and nutrition, household income and expenditure, and labour force experience.^14^

## Demographics and Labour Force

This section documents demographic changes and labour informality in emerging Asian economies. The focus is on Indonesia – the largest Southeast Asian economy and the fourth most populous country in the world, but we also include data on other major economies in East Asia (EA) and Southeast Asia (SEA). First, using the UN population data from 1950 to 2100 (UN, 2019), we first present past demographic trends and projections for Indonesia and selected major EA and SEA economies.^15^ The second part of this section then deals with Indonesian labour force, using the IFLS data. In that part, we outline the composition of the Indonesian labour force, labour earnings and sectoral transitions.

### Demographic Change

**Fig. 2:**
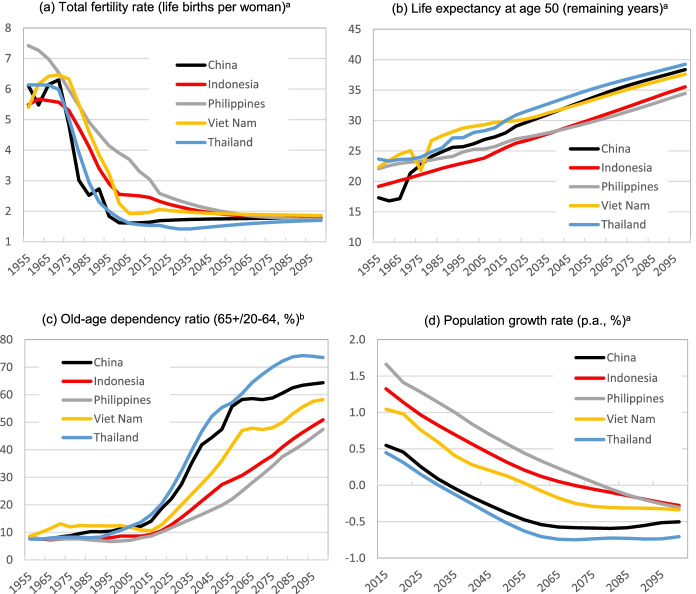
Demographic drivers and population ageing in emerging Asia*. *Notes*: *Based on UN, 2019; ^a^For 5-year average with displayed last year (of the interval) (e.g. 1955 representing 1950–1955); ^b^For the given year (e.g. 1955)

The UN population data and projections allow comparisons of the demographic transitions that emerging Asian economies have been and will be experiencing, based on UN (2019). The selected countries include Indonesia, China, Philippines, Vietnam and Thailand. We first discuss demographic drivers (i.e., changes in fertility and life expectancy implied by survival probabilities). Then, we present key demographic outcomes for old age dependency ratio and population growth. These demographic inputs and outcomes for the five selected countries are plotted in Fig. 2.

#### Fertility and Survival Rates

Changes in the age distribution, size and growth of Asian populations have been and will be shaped by changes in fertility and life expectancies implied by survival rates. The changes in the total fertility rate and life expectancy at age 50 in the selected emerging Asian economies over the period of 1950–2100 are depicted in Fig. 2a and b, respectively.^16^

Figure 2a shows that the total fertility rates (TFR) (i.e., live births per woman of reproductive age) have experienced pronounced declines in the displayed countries in the past. In Indonesia, the TFR has declined to just over 2 births per woman in 2020, from the peak TFR of over 5.5 births in the 1960s.^17^ In Vietnam, the decline in TFR was even more substantial, from 6.2 births per woman in 1955–60 to currently about 2.1. Similar developments were seen in China and other emerging Asian economies. Notice that these declines are much more pronounced than those observed in developed regions (comprising Europe, Northern America, Australia/New Zealand and Japan in UN (2019)), where average TFR was “only” 2.8 births per woman in 1955–60 (less than half of those in many EA and SEA countries), but their current TFRs are not that different from those in the displayed Asian economies. Furthermore, many emerging SEA economies are projected to undergo further declines in the TFR over the course of this century. For instance, the Indonesian TFR is projected to further decline to 1.8 births by 2100 (under the UN base case projection scenario).

Figure 2b displays past development and future projections for average life expectancy at age 50, implied by survival probabilities (averaged over both sexes) at older ages. As shown, this demographic measure has been increasing in the selected countries in the past, with increases projected to continue, but even at exponential rates in these countries over the course of this century. For example, average life expectancy at age 50 in Indonesia was about 19 years in 1950–55, whereas it is projected to almost double to over 35 years (age 85) in 2100, with most of the increase projected in the period of 2020 to 2100. Similar projections are shown for other emerging Asian economies, with life expectancies at age 50 almost reaching 40 years in China and Vietnam by 2100. Note that similar life expectancy (at that age) is projected for developed regions (e.g. the US) by the end of the twenty-first century.^18^

#### Population Estimates and Projections – Population Ageing

To demonstrate pronounced population ageing in the EA and SEA regions, Fig. 2 also displays UN estimates and projections for (2c) the old-age dependency ratio and (2d) the annual growth rate of the total population. The projections are based on the medium fertility variant, with fertility and survival rates (and the implied life expectancy) discussed above.

The old-age dependency ratio is defined in this paper as the percentage of the population aged 65+ relative to the working age population aged 20–64.^19^ As shown in Fig. 2c, the old-age dependency ratio in Indonesia has increased only slightly in the last 60 years to just over 10% in 2020, but the ratio is projected to increase by a multiple of 5 (to about 50%) by 2100. These dramatic changes in this dependency ratio are due mainly to projected increases in elderly populations but also due to declines in (growth rates of) working age populations. In China, the old-age dependency ratio has more than doubled over the last 20 years and is expected to further increase by a multiple of 3 to over 64% by 2100. It is projected to be even higher than the average ratio of developed regions in 2100 (UN, 2019). The displayed SEA economies (such as Indonesia and Vietnam) have experienced much smaller increases in the old-age dependency ratio to date than China has, but they will face much larger increases over the course of this century.^20^

As shown in Fig. 2d, population ageing will be accompanied by declining population size, with the population growth rates projected to decline and be negative.^21^ Although many of these populous regions have experienced a much higher population growth in the past (compared to developed regions) driven by very high TFRs, the future population growth rates will become negative in coming years. In Indonesia and Vietnam, their populations are projected to start declining in the second half of this century, while the total population in China will start declining after 2030 (based on UN, 2019). As shown, Thailand is projected to experience largest percentage population declines, with over −0.7% growth rates in the second half of this century.

Such pronounced demographic changes in Indonesia and other emerging Asian economies over the course of this century will have vast economy-wide implications. In emerging Asian economies and particularly in SEA, these demographic changes are occurring simultaneously with other challenges such as high and persistent labour informality (discussed in the next subsection) and the challenges specific to older adults (discussed in Section 5). Importantly, these demographic changes (i.e., fewer adult children supporting their parents and greater expected lifespans with survival improvement particularly at older ages) highlight the urgency of developing a formal social policy (that is fiscally sustainable) to avert large-scale poverty among older cohorts.

It should be noted that the data and projections reported in UN (2019) have been uncritically accepted in this analysis. While the accuracy of these data may be challenged, for the purposes of international comparison it is convenient to draw on data sets that have been coordinated across countries. National data and projections may yield somewhat different outcomes.

### Labour Force

This section focuses on the labour force in Indonesia. Of particular concern are trends in skill type, and the workforce split between formal and informal employment. Skill development and formalisation are critical inputs into productivity improvements and growth, which in turn will provide the revenue flows necessary to finance social protection for the elderly. The major data source used here is IFLS – the 2000, 2007 and 2014 waves.

The focus is on the labour force of working age population aged 20–54, reporting on (i) labour force composition (accounting for formal and informal (and low and high skill) workers) and (ii) their earnings over the working lifecycle.

A common feature across emerging Asian economies is very high (and persistent) informal employment, with cross-country analysis reported, for example, by ILO (2018a, b).^22^ In this section, we document very high informal employment in Indonesia, using the IFLS data that allow us to capture differences by age, skill type and many other factors.

Below, we briefly discuss data selection, relevant to this section, followed by presenting the key observations for the Indonesian labour force.

#### Data Selection

As discussed in Section 2.2, we define “informal” workers (or employment) as those who do not receive any health insurance and medical benefits from their employers. For the empirical analysis in this sub-section, specific restrictions for the output sampling are applied. These include the following:

- We use data for male workers only. The main reason for this restriction is the labour market participation in developing countries, where women are less likely to have a continuous job compared to men. Note that the data for Indonesia shows that the main reason for women never having worked or having suspended their work is marriage or taking care of their children.^23^
- We restrict the age of workers to be in the range from 20 to 54 years (because of the formal sector retirement age in Indonesia that has been set at 55 years during most of the period covered by the employed IFLS data^24^) and keep the below information at the individual level (for males) for age, education, employment status, earnings variables and transition probabilities.
- We keep farmers and the self-employed in the sample since they are very populous groups in developing countries.
- People with no jobs (6.6% of the total number of interviews across the three IFLS waves conducted in 2000, 2007 and 2014). In addition, people who report themselves as “unpaid family workers” (3.6%) are excluded from the empirical analysis presented in this subsection.
- People are defined as “high” skilled only if they completed senior high school.^25^
- All observations with missing information are also removed from the constructed sample for which (each wave separately and all three waves combined) we report the number of observations in Table 1.

**Table 1:** Composition of employment (males aged 20–54)

Employment type^a^	Skill type^b^	2000	2007	2014	Overall^c^
Formal	Low	6.0%	3.9%	5.4%	5.1%
	High	16.0%	16.1%	19.8%	17.6%
	(Total)	22.0%	20.0%	25.2%	22.6%
Informal	Low	56.1%	49.9%	43.2%	48.8%
	High	21.9%	30.1%	31.7%	28.6%
	(Total)	78.0%	80.0%	74.8%	77.4%
No. of observations^c^		7041	8866	10,646	26,553

^a^See Section 2.2 for the informal employment definition used

^b^Based on educational attainment, with “High” depicting those who completed 12 years of schooling; ^b^Derived from all three waves - IFLS in 2000, 2007 and 2014

^c^Number of observations used in each displayed IFLS wave (either 2000 or 2007 or 2014) and combined across all three waves in column “Overall”

#### Composition of Employment – Formal vs. Informal

The composition of the Indonesian labour force (based on data for males aged 20–54, with the data selection outlined above) is provided in Table 1 for all three IFLS waves and also using each wave separately, in order to document the recent trends. In the table, we decompose the labour force into four types, based on their employment type and skill (or educational) type, both defined above.

Several key observations can be drawn from Table 1. First, focusing on the results in the column “Overall”, the share of the informal sector is high, 77.4% of total employment (of males aged 20–54).^26^ Second, most of these formal workers are high skill (with completed 12 years of schooling) – 17.6% of the sample, while around 5.1% of the labour force is of formal low skill type. This amounts to 22.6% of the sample in formal employment. In contrast, most of informal employment is in the low skill (with not-completed high school) category, which is the largest and amounting to almost 50% of working age population (48.8% in the three IFLS waves with 26,553 observations).^27^

Third, when comparing the results from the different IFLS waves, there has been a (rather small) decline in informal employment by 3.2 percentage points (p.p.) between 2000 and 2014.^28^ In fact, as shown in Table 1, the informal share has increased by about 2 p.p. between 2000 and 2007, to 80% of the sample (of men aged 20–54). This then demonstrates the “persistent” property of informal employment. Fourth, the skill composition of the labour force, particularly of informal workers has changed significantly in that period of 2000 to 2014. Specifically, the share of the informal high skill type has increased by 10.1 percentage points in 2014, when compared to 2000. Note that the share of high skill (operating in both formal and informal employment) has increased to over 50% of the sample in 2014, from about 38% in 2000. Distinguishing between different cohorts (which is not shown here but has been done and could be requested), the increase is particularly significant for young cohorts – gaining educations but many remaining in informal employment.^29^

#### Lifecycle Labour Earnings

Above we have shown persistently large informal employment in Indonesia. In this subsection, we document labour earnings of the sample over the lifecycle. Specifically, we report annual labour earnings of the four (employment and skill) types of workers (as defined above). For each individual, the labour earnings variable is constructed as the total of salary (bonus included) from the main job and extra jobs (if any) and the net profit of business from their own farm or non-farm business.^30^ By nature, a formal worker is mainly a wage worker, while an informal worker is usually self-employed. Note that for an informal worker, as they only report the net profit from their business (or their household business), it is difficult to distinguish between labour earnings and capital earnings. Therefore, we use the net profit in the calculations of their earnings in full. When pooling up data from different survey years, the Consumer Price Index (CPI) obtained from the International Financial Statistics (IFS) is used to construct real annual earnings.^31^

**Fig. 3:**
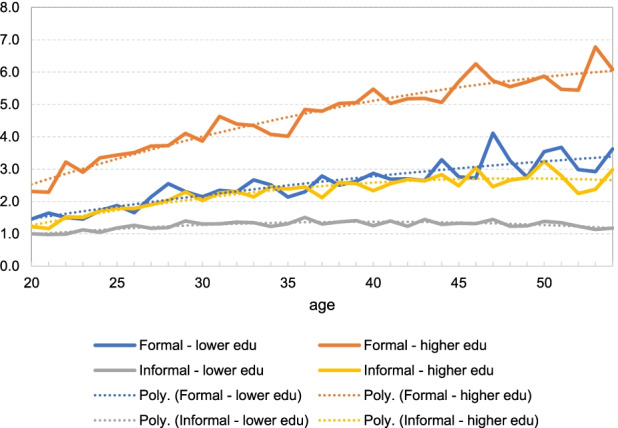
Normalised average earnings profiles. *Note*: Using IFLS 2000–2014, we calculate log of annual earnings (in IDR) for each employment and skill type and then normalise these by log earnings of informal low-skill type aged 20

In Fig. 3, we plot labour earnings (ln of annual male earnings across the three IFLS waves) over the working years 20–54, with all the profiles normalised by earnings (ln of annual labour earnings) of the informal low type aged 20 (=15.58). The objective is to provide comparison of lifecycle earnings across the four types of workers. As shown, individuals in formal employment have significantly higher earnings compared to those operating in the informal employment. Moreover, the slope of the earnings profile for formal workers is steeper over early working years, increasing more significantly than for informal workers, who, on average, experience gradually declining earnings at older ages. The skill type also matters. Comparing those with different skills in the same sector show that high skill significantly more than those with low skill (who has not completed high school). For example, the figure shows that the formal high type workers in age group 50–54 earn on average about six time more than the informal low type workers of the same age group.

#### Summary

Our empirical investigation (based on the IFLS) has shown that informal employment in Indonesia is persistently large, at almost 80% of total employment. While most formal workers are high skill (with 12 years of schooling), most informal workers are shown to be in the low skill category. In the period of 2000 to 2014, more workers gain education (the share of high skill workers has increased), but that has not resulted in a significant increase in the share of formal employment. We have also shown that those operating in formal employment have significantly higher labour earnings, with formal high skill earning, on average, 6 times more than informal low skilled workers. They also experience a much higher growth in labour earnings over the lifecycle. Hence, the gap between labour earnings of formal and informal workers widens by age.

In Kudrna et al. (2020), we have also calculated employment transitions (comparing IFLS waves in 2007 and 2014). The results indicate that only 11% of informal workers (observed in 2007), moved to formal employment in 2014, while about 30% of formal workers transitioned to informal employment. Comparing cohort-specific results shows that the probability of transitioning from formal to informal has a u-shaped (high for younger and particularly higher older cohorts), whereas the share of informal workers who moved to formal employment declines by age. For the high formal to informal transition at older ages, this is due to the formal retirement (pension access) age. Importantly, those transitioning from formal to informal employment, on average, earned significantly lower income than those staying in formal employment. The significantly higher earnings in formal employment seem to support the ILO’s view “that most people enter (or transition out of formal employment to) the informal economy not by choice, but as a consequence of a lack of opportunities in the formal economy and in the absence of other means of livelihood” in developing countries.^32^^,^^33^

As for the international comparison, Kudrna et al. (forthcoming), drawing on ILO (2018b) data, show that similar issues, documented above for Indonesian labour force (e.g., high informality), apply to other emerging economies in EA and SEA countries, with informal employment in some countries even exceeding 90% (e.g. in Cambodia and Lao PDR).

## Older People

This section documents living arrangements, employment rates and labour supply, and income sources of older adults, with the results presented for people in Indonesia aged 50 years and over. The results are drawn from the IFLS waves 2000–2014, with more detailed results provided in tables and figures placed in the Appendix.

### Marital Status and Living Arrangements

**Fig. 4:**
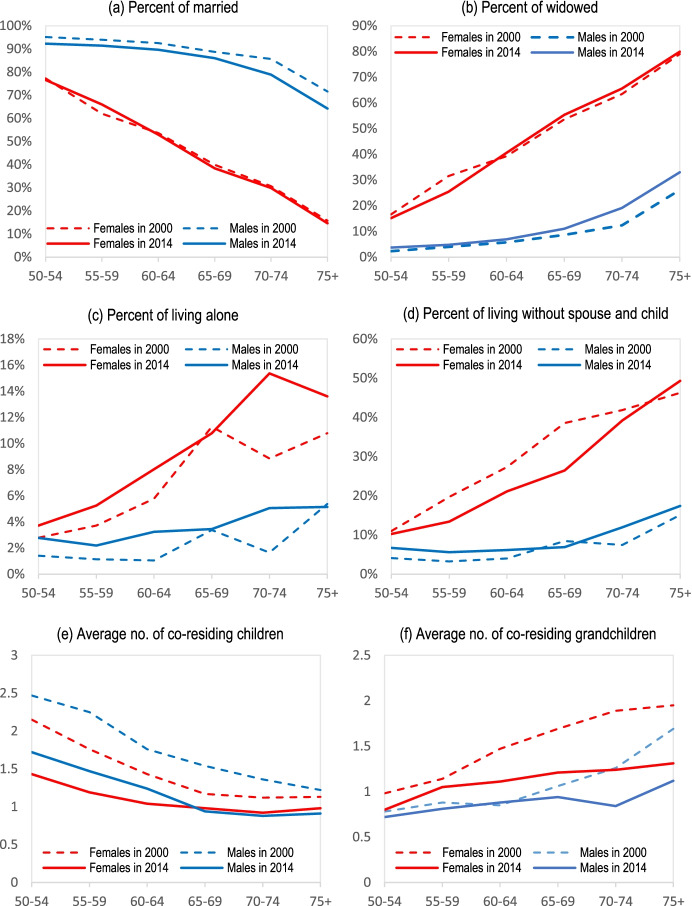
Marital status and living arrangements at older ages. *Source:* IFLS 2000 and 2014

The marital status and living arrangements of older adults are depicted in Fig. 4. Marital status itself has important implications for their living arrangements. As shown in Fig. 4a and b, while most older men are married (over 86% in the 65–69 age group in 2014), the share of widowed women is significantly higher (55% in the 65–69 age group in 2014) and it increases with age (almost 80% in the 75+ age group in 2014). These observations reveal that older women might be more vulnerable in terms of not having informal support from their spouse, compared to older men.

Figure 4c and d report living arrangements of older women and men in Indonesia, and changes in these arrangements between 2000 and 2014. It confirms the importance of the family for old-age support. Most older adults live with their children, especially women.^34^ While only a small fraction of older adults live alone, the proportion with no spouse and no child is large and has been increasing, particularly in the case of older women.

However, the probability of co-residing with children decreases with the age of the older adults as well as over time, as shown in Fig. 4e and f depicting the average number of co-residing children and grandchildren, respectively. For example, older women aged 75+ shared the residence on average with 1.13 children and 1.95 grandchildren in the 2000 wave, but only with 0.98 children and 1.31 grandchildren in the 2014 wave (due probably to regional migration).

### Employment and Labour Supply

**Fig. 5:**
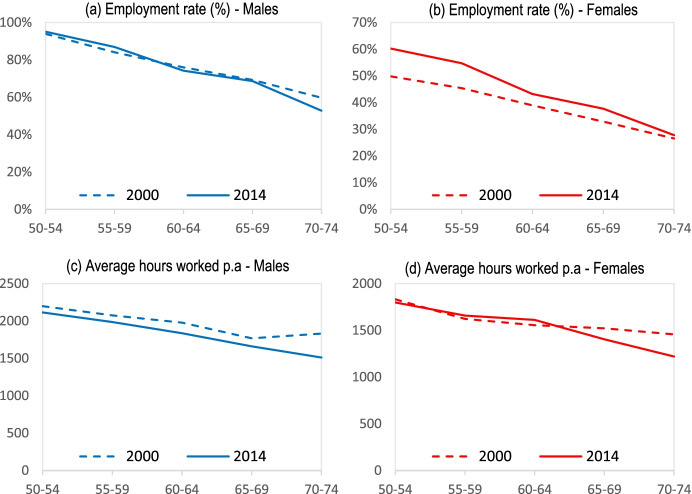
Employment and labour supply at older ages. *Source:* IFLS 2000 and 2014

This sub-section focuses on employment and hours worked by older adults in Indonesia, both males and females, reporting on their employment rates, types of jobs (part-time or full-time) and working hours at older ages. Based on ILFS waves in 2000 and 2014, the results are depicted in Fig. 5.

Figure 5a and b compare the changes in employment rates in different survey years for different age groups of older adults. We define employment as workers who report working in a paid job. As shown, the employment increase was large particularly for women aged 50–54 after 2000. Increasing labour supply at older ages for women is similar to the observations in developed countries, but the level of male employment and the gap between male and female employment rates are both much higher than in developed countries.^35^ Note that similar findings are reported by Cameron and Cobb-Clarke (2002), showing that many Indonesians continue to work well into old age, using the 1993 IFLS data. As already discussed, we focus on the more recent IFLS surveys.^36^

Labour supply of men and women at older ages is depicted by their average annual hours worked in Fig. 5c and d. As shown, not only the employment rate but also working hours are high at older ages, particularly for men. For example, most working men aged 60 to 64 are working almost 2000 h per year and those 65+ over 1500 per year. Compared to early survey years, the labour supply has decreased (in particular, at very older ages) in more recent surveys but the hours are much higher than those observed in developed countries.^37^

### Total Income and Income Sources

**Fig. 6:**
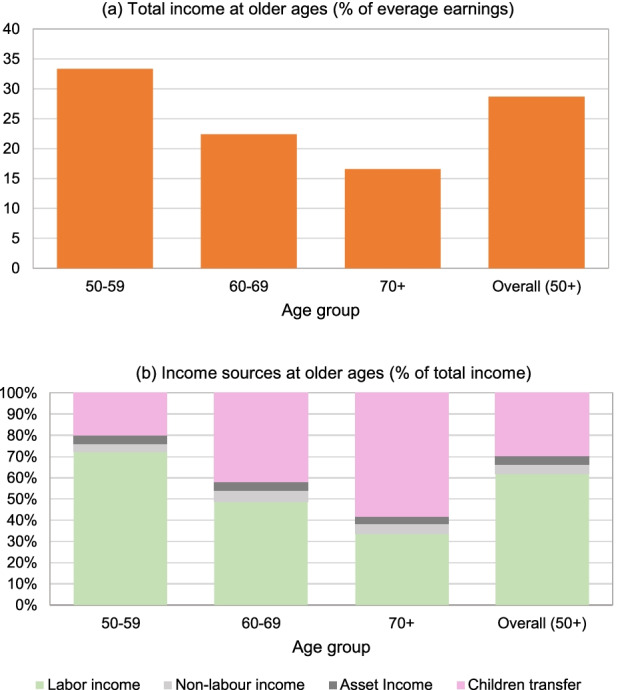
Total income and income sources at older age. *Source:* IFLS 2000–2014

We now report the total income and income sources of older adults in Indonesia (those aged 50 years and over). The four main sources of income that we distinguish are labour income (including farm and non-farm income), non-labour income (including pension, insurance money, and lottery), assets income (including income from savings, and rent), and private transfers from children. As opposed to the previous subsection on employment of older adults, here we use data set and responses by household head (on her/his income and income source) and we do not distinguish by gender. The IFLS observations (combined and CPI adjusted from waves in 2000, 2007 and 2014) for total income and income sources are provided in Fig. 6. These observations relate to household head only, not to the whole household.^38^ In Fig. 6a, the total income is presented as a percentage of average earnings (of males aged 20–54 in formal employment), while the income sources in Fig. 6b are given as a percentage of that total income.^39^

Several observations can be drawn for Fig. 6. First, total income (per household head) at older ages is low. For the overall age group 50+ it is shown to be less than 30% of average earnings. Second, this “replacement rate” measure declines for older age groups. As indicated, in the overall sample (across the three waves), the average total income of age group 50–59 is 33.4% of average earnings, while for those 70+, average total income is 16.6% of average earnings – less than half of the replacement rate for those 50–59.

Third, labour income is the most important source of total household income for those 50+, about 61% of total income due to labour income, as indicated in Fig. 6b. It is an important source of total income particularly for younger cohorts of older adults, amounting to over 72% of total income of cohort 50–59 across the three IFLS waves. Fourth, the reliance on labour income declines with age, with private transfers from children playing an important role as an income source as people age. As shown by Fig. 6b, private transfers (i.e., net transfers from children) account for less than 20% of the total income for people aged 50–59 in 2000 and 2014. However, this within-family financial support becomes more crucial for people at a higher age, with those aged 70+ deriving more than 55% of their income from private transfers.

In contrast to developed countries where transfers typically flow from parents to their children via bequests, in developing countries, transfers from children play an important role for sustaining consumption of older adults. As shown, in Indonesia, a majority of the old-age population does not receive a public pension as a result of a large informal economy. However, more than 70% of those aged 70+ receive positive net money transfers from their children (see Table 5).^40^

### Summary

Our analysis reveals that most older adults in Indonesia share residence with their children and the share of older adults co-residing with their children increases with age and is particularly high for older women. While employment rates for men and women decrease after the mandatory retirement age (56 in 2014), many older adults continue to work, with both employment rates and hours worked remaining high, in particular for males. The most important source of income for older adults is labour income. And private (within-family) transfers become a more important source of income as people age. Similar findings are reported by World Bank (2016, 2020b) for older adults in Indonesia (using other data sources) and other emerging Asian economies.

## Concluding Remarks

This paper documents the socio-economic circumstances of older adults in Indonesia. Older cohorts will become relatively more numerous in coming decades, as declining fertility and increasing mature age life expectancy shape a changing demographic structure for the country. It identifies trends in the labour force for younger cohorts, labour force participation, skill development, and the formal-informal labour force split. Increasing skills and formalisation formalisation will be critical for growth and revenue flows in coming decades, and therefore for sustainable social protection policies.

This demographic transition, combined with other social and economic forces associated with Indonesia’s economic growth, highlights the importance of the country’s social protection policy structure, especially as it is directed towards older cohorts. Our analysis shows that social protection is very undeveloped in Indonesia. Poverty among older age groups is very high: pensions are not available to more than 75% of the older population. Neither is there any likely prospect of this circumstance correcting itself through growth. While Indonesia has grown substantially through this century, recording an average of more than 5% per annum up to the onset of the pandemic, the proportion of workers in the informal sector has remained persistently high, at close to 80%.

Our analysis reveals that most older adults in Indonesia share residence with their children and the share of older adults co-residing with their children increases with age and is particularly high for older women. While employment rates for men and women decrease after the mandatory retirement age (56 in 2014), many older adults continue to work, with both employment rates and hours worked remaining high, in particular for males. The most important source of income for older adults is labour income. And private (within-family) transfers become a more important source of income as people age. Similar findings are reported by World Bank (2016, 2020b) for older adults in Indonesia (using other data sources) and other emerging Asian economies.

While most formal workers are classified as high skill (with 12 years of schooling), most informal workers are shown to be in the low skill category. In the period 2000 to 2014, education policy has led to more young workers being in the high skill category, but this has not resulted in a significant increase in the share of formal employment. We also find that that those operating in formal employment have significantly higher labour earnings, with formal high skill earning, on average, 6 times more than informal low skilled workers. They also experience a much higher growth in labour earnings over the lifecycle. Hence, the gap between labour earnings of formal and informal workers widens by age.

In Kudrna et al. (2020), we have also calculated employment transitions (comparing IFLS waves in 2007 and 2014). The results indicate that only 11% of informal workers (observed in 2007), moved to formal employment in 2014, while about 30% of formal workers transitioned to informal employment. Comparing cohort-specific results shows that the probability of transitioning from formal to informal has a u-shaped (high for younger and particularly higher older cohorts), whereas the share of informal workers who moved to formal employment declines with age. The high formal to informal transition at older ages is due to the formal retirement (pension access) age. Those transitioning from formal to informal employment, on average, earned significantly lower income than those staying in formal employment. The significantly higher earnings in formal employment seem to support the ILO’s view “that most people enter (or transition out of formal employment to) the informal economy not by choice, but as a consequence of a lack of opportunities in the formal economy and in the absence of other means of livelihood”.^41^^,^^42^

These features of the economy and the profile of older cohorts suggest that a major effort will be required over the next two decades to construct and effectively implement substantial and sustainable policy social protection structures, if widespread extreme hardship is to be avoided. This is a major challenge, requiring not only a statistical portrait of the country’s labour force and older population, but also a consistent economic framework within which to interrogate alternative social protection designs. The work reported here has been undertaken to help parameterise a major long run macroeconomic model, incorporating demographic transition, to inform such a perspective and generate quantitative estimates of the impacts of alternative policy packages.

As we have suggested above, Indonesia is not alone in confronting these challenges. Kudrna et al. (forthcoming), drawing on ILO (2018b) data, show that similar issues, documented above for Indonesian labour force (e.g., high informality), apply to other emerging East and South-east Asian countries, with informal employment in some countries exceeding 90% (e.g. in Cambodia and Lao PDR).

## Figures and Tables

**Fig. 7:**
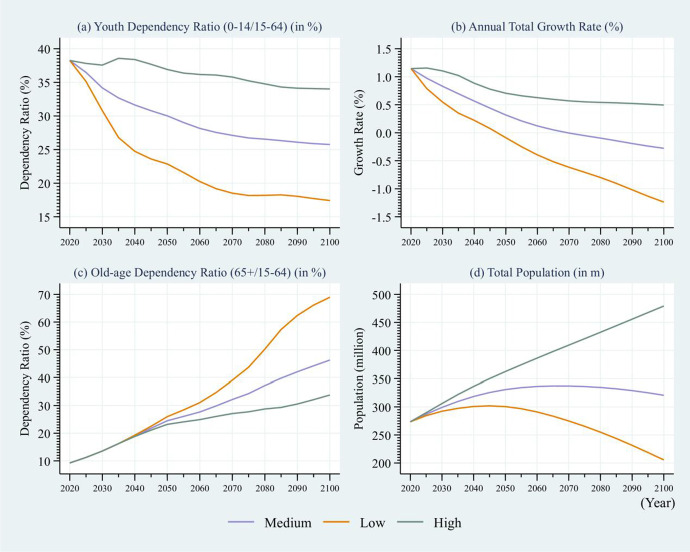
Alternative demographic projections for Indonesia. *Source*: United Nations (2019)

**Fig. 8:**
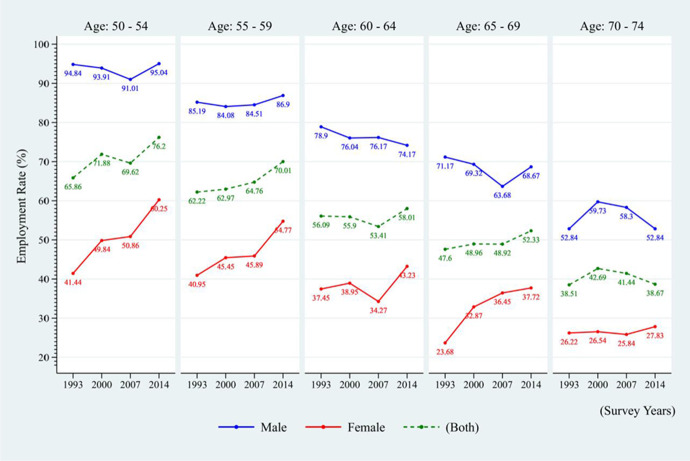
Employment rate at older ages. *Source:* Authors’ calculation from IFLS 2000–2014

**Fig. 9:**
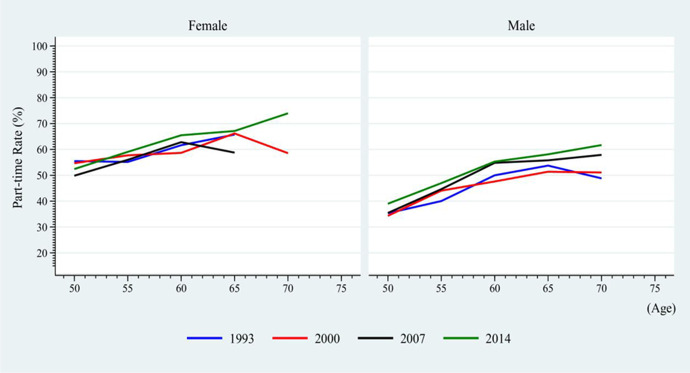
Percentage of working part-time at older age. *Source:* Authors’ calculation from IFLS 1993–2014

## Appendix 1

### Demographic Projections for Indonesia

Here, we focus on the (alternative) projections for Indonesia (UN, 2019), with the alternative population projections compared to the medium (most likely) scenario, already discussed in Section 3.1). Figure 7 plots two alternative (low and high fertility) and base-case projections for old-age dependency ratio and population growth, and also for the total population and youth dependency ratio (under different fertility assumptions). The base case or medium projections are based on medium assumptions for fertility and survival rates, depicted in Fig. 2 in the text. As shown, assumed fertility rates have significant impacts on the total population size, growth and its age distributions, with substantial differences in the presented demographic outcome variables in 2100 when comparing low- and high-fertility projections. For example, the figure shows that under low fertility scenario, old age dependency ratio would be more 35 percentage point higher and the total population would fall by over 270 million in 2100, compared to what these variables would be under the high-fertility projection scenario. These alternatives also indicate that the declining TFR in the past will be driving the population ageing in Indonesia in years to come, whereas improvements in survival probabilities will cause the lifespan to extend, particularly at older age.

This appendix also provides further data on older adults and their marital status and living arrangements, employment and their income sources, with these detailed results taken from Kudrna et al. (2020).

### Further Observations for Marital Status and Living Arrangements at Older Age

Tables included in this subsection provides further details of marital status and living arrangement of older males and females, with the results based on the IFLS waves 2000, 2007 and 2014. Specifically, Table 2 provides marital characteristics by year, age group and gender (i.e., percentage in each category – married and others for Females and Males). Then, Table 3 shows living arrangements by year, age range and gender as a percentage of those living alone or with spouse and/or children. Table 4 reports on the average number of co-residing children and grandchildren, as depicted in Fig. 4e and f in the main text, but here also in IFLS Wave 2007.

**Table 2:** Older adults and marital status

Year	Age Range	Female					Male				
		Unmarried (%)	Seperated (%)	Divorced (%)	Widowed (%)	Married (%)	Unmarried (%)	Seperated (%)	Divorced (%)	Widowed (%)	Married (%)
2000	50–54	1.77	0.63	3.8	16.58	77.22	1.15	0.26	1.15	2.3	95.15
	55–59	1.2	0.96	4.32	31.53	61.99	1.13	0.28	0.7	3.94	93.95
	60–64	0.98	1.13	5.06	39.24	53.59	0.17	0.35	1.21	5.72	92.55
	65–69	0.75	1.32	4.51	53.57	39.85	0.24	0.48	1.92	8.65	88.7
	70–74	0.24	0	5.74	63.4	30.62	0	0.28	1.65	12.4	85.67
	75-	1.18	0.98	3.14	79.02	15.69	0.77	0	1.53	26.09	71.61
2007	50–54	3.03	1.18	4.63	19.55	71.61	1.23	0.28	1.7	2.17	94.62
	55–59	1.84	0.65	4.11	28.46	64.94	0.7	0.12	0.93	4.54	93.71
	60–64	1.67	0.7	5.43	43.04	49.16	0.82	0.16	0.98	6.69	91.35
	65–69	1.43	0.72	3.16	51.65	43.04	0.71	0	1.24	10.42	87.63
	70–74	1.4	0.7	2.56	67.13	28.21	0.29	0.86	2.3	16.09	80.46
	75+	1.12	0.64	2.71	80.06	15.47	0.22	0.88	1.53	30.42	66.96
2014	50–54	2.22	1.17	4.9	15.14	76.57	1.72	0.6	1.72	3.67	92.28
	55–59	2.5	0.97	5.08	25.42	66.02	1.71	0.19	1.9	4.75	91.45
	60–64	1.75	0.21	4.53	40.58	52.94	0.89	0.22	2.35	6.94	89.6
	65–69	1.43	0.16	4.75	55.31	38.35	0.86	0.35	1.73	11.05	86.01
	70–74	0.84	0.51	3.21	65.54	29.9	0.46	0	1.61	19.08	78.85
	75+	0.57	0.28	4.67	79.89	14.59	0.2	0	2.57	33	64.23

IFLS 2000–2014, Authors’ calculation

**Table 3:** Older adults and living arrangements

Year	Gender	Age range	No. of obs.	Living arrangement (%)				
				With spouse and child	With spouse and no child	No Spouse but with child	No spouse and no child	
							Alone	With others
2000	Female	50–54	790	48.86	25.82	14.3	2.78	8.23
		55–59	834	32.73	27.22	20.38	3.72	15.95
		60–64	711	20.96	30.66	21.1	5.77	21.52
		65–69	532	13.35	25.75	22.37	11.28	27.26
		70–74	418	11.24	17.94	28.95	8.85	33.01
		75+	510	3.33	10.98	39.41	10.78	35.49
	Male	50–54	783	78.03	15.33	2.55	1.4	2.68
		55–59	711	74.4	18.42	3.94	1.13	2.11
		60–64	577	64.82	26.69	4.51	1.04	2.95
		65–69	416	51.68	35.34	4.57	3.37	5.05
		70–74	363	51.52	33.61	7.44	1.65	5.79
		75+	391	34.02	35.81	15.09	5.37	9.72
2007	Female	50–54	1187	49.2	20.3	18.7	4.04	7.75
		55–59	924	36.15	27.16	21.1	6.06	9.52
		60–64	718	22.14	26.18	26.6	9.47	15.6
		65–69	697	16.21	25.68	28.98	10.47	18.65
		70–74	429	11.19	15.62	32.87	15.85	24.48
		75+	627	4.94	10.05	41.63	14.83	28.55
	Male	50–54	1060	75.94	16.89	2.74	2.08	2.36
		55–59	859	67.52	23.86	4.07	2.1	2.44
		60–64	613	61.66	27.41	5.71	2.12	3.1
		65–69	566	52.65	33.57	7.6	2.65	3.53
		70–74	348	43.1	37.07	8.33	4.6	6.9
		75+	457	35.67	29.76	20.79	4.81	8.97
2014	Female	50–54	1532	55.68	18.47	15.6	3.72	6.53
		55–59	1239	39.79	24.7	22.11	5.25	8.15
		60–64	971	28.42	23.07	27.39	8.03	13.08
		65–69	631	15.21	22.82	35.5	10.78	15.69
		70–74	592	10.47	18.75	31.59	15.37	23.82
		75+	706	4.39	10.2	36.12	13.6	35.69
	Male	50–54	1335	71.39	17.98	3.97	2.77	3.9
		55–59	1053	63.53	26.31	4.56	2.18	3.42
		60–64	894	56.6	30.54	6.71	3.24	2.91
		65–69	579	46.63	36.79	9.67	3.45	3.45
		70–74	435	39.08	38.62	10.34	5.06	6.9
		75+	506	28.46	34.98	19.17	5.14	12.25

IFLS 2000–2014, Authors’ calculation

**Table 4:** Older adults and average number of co-residing children

Year	Age range	Average number of co-residing children			
		Female		Male	
		Children	Grandchildren	Children	Grandchildren
2000	50–54	2.15	0.98	2.47	0.78
	55–59	1.76	1.14	2.25	0.88
	60–64	1.43	1.47	1.76	0.85
	65–69	1.17	1.69	1.54	1.06
	70–74	1.12	1.89	1.36	1.26
	75+	1.13	1.95	1.22	1.69
2007	50–54	1.64	0.83	2.07	0.64
	55–59	1.36	0.96	1.6	0.65
	60–64	1.18	1.03	1.45	0.85
	65–69	1.04	1.29	1.15	0.91
	70–74	1.07	1.36	0.95	0.91
	75+	1	1.39	1.03	1.1
2014	50–54	1.43	0.8	1.72	0.72
	55–59	1.19	1.05	1.47	0.81
	60–64	1.04	1.11	1.24	0.88
	65–69	0.98	1.21	0.94	0.94
	70–74	0.92	1.24	0.88	0.84
	75+	0.98	1.31	0.91	1.12

IFLS 2000–2014, Authors’ calculation

### Further Observations for Employment at Older Age

The figures included in this subsection provides further details of employment of older males and females, with the results derived from the IFLS waves 1993, 2000, 2007 and 2014.

Figure 8 shows the employment rates of working men and women at older ages (5-year age groups) in different survey years of 1993, 2000, 2007 and 2014. As expected, the employment rate declines over the remaining life cycle. Note that at the time of IFLS wave 5 (2014), the retirement age in Indonesia (for both employees in the private sector and public servants) was 56 (Muliati & Wiener, 2014). This (formal retirement age) effect is depicted by the decline in the employment rate for the age group 55–59. The figure reveals that male workers’ employment rate decreases after the retirement age.

Figure 8 also shows significant increases in the employment rate for older females in more recent survey years. Except for the decrease in 2007, due to the global financial crisis, the female employment rate is significantly higher for all age groups in more recent years, and particularly in 2014, as depicted by the green line in Fig. 5 (in the text) for females.

The labour force data also reveals interesting patterns in the part-time and full-time work types, depicted by Fig. 9 showing the proportion of part-time workers by age and gender in different survey years. A part-time worker is defined as one who works less than 30 h per week in their main job. It should be noted that in developing countries, the proportion of informal workers is large, and they often work more than one job in the same period or work more intensively in some seasons. Therefore, using the main job to define part-time workers might underestimate the total number of working hours per week. We therefore use a different definition focusing on annual total working hours. Following Blundell et al. (2016), we consider part-time workers as those who work less than 1500 h per year (which is equivalent to working no more than 30 h per week, assuming 50 weeks per year).

Overall, Fig. 9 shows that part-time employment increases with age and is more prevalent in women. When comparing the employment rates between 2000 and 2014, the rate was higher in 2014 for all age groups of both women and men. There was no clear trend in 2007 compared to other survey years, most likely due to the global financial crisis during which the labour market fluctuated dramatically.

### Further Observations for Income Sources at Older Age

Additional details for income sources at older age for the three latest waves of IFLS (2000, 2007 and 2014) are presented in Tables 5 and 6.

**Table 5:** Income sources of older adults (Percentage with given income source)

Year	Age range	No. of obs.	Income sources (%) (Household head only)^a^						Net transfer from children^b^		
			Labor income		Non-labor income		Non-labor income				
			Yes	No	Yes	No	Yes	No	(−)	0	(+)
2000	50–59	808	61.88	38.12	16.09	83.91	7.43	92.57	19.43	33.66	46.91
	60–69	396	49.24	50.76	15.4	84.6	6.57	93.43	9.6	31.31	59.09
	70+	164	36.59	63.41	11.59	88.41	6.71	93.29	5.49	34.76	59.76
	All (50+)	1368	55.19	44.81	15.35	84.65	7.09	92.91	14.91	33.11	51.97
2007	50–59	922	59	41	0.43	99.57	4.77	95.23	13.88	29.83	56.29
	60–69	416	44.23	55.77	1.44	98.56	4.33	95.67	9.86	18.99	71.15
	70+	129	35.66	64.34	0	100	3.1	96.9	1.55	24.03	74.42
	All (50+)	1467	52.76	47.24	0.68	99.32	4.5	95.5	11.66	26.24	62.1
2014	50–59	1136	68.57	31.43	5.02	94.98	10.04	89.96	20.77	24.91	54.31
	60–69	442	52.94	47.06	9.05	90.95	10.41	89.59	14.03	16.97	69
	70+	134	35.82	64.18	4.48	95.52	7.46	92.54	6.72	18.66	74.63
	All (50+)	1712	61.97	38.03	6.02	93.98	9.93	90.07	17.93	22.37	59.7

IFLS 2000–2014, Authors’ calculation

^a^% receiving/not-receiving given income

^b^% receving negative, zero or positive net tranfer from their children

**Table 6:** Distribution of income sources (Percentage of total household income)

Year	Age range	No. of obs.	Income sources (%) (Household head only)			
			Labor income	Non-labour income	Asset Income	Children net transfer
2000	50–59	808	66.26	10.29	4.53	18.92
	60–69	396	51.59	9.55	4.02	34.84
	70+	164	35.18	8.22	4.15	52.46
	All (50+)	1368	58.51	9.84	4.34	27.3
2007	50–59	922	63.64	0.04	4.38	31.93
	60–69	416	44.37	0.59	1.96	53.08
	70+	129	31.34	0	2.17	66.49
	All (50+)	1467	55.44	0.2	3.5	40.86
2014	50–59	1136	82.63	2.12	3.54	11.7
	60–69	442	55.13	12.56	5.28	27.03
	70+	134	36.27	4.44	5.19	54.09
	All (50+)	1712	71.99	5.01	4.12	18.88

IFLS 2000–2014, Authors’ calculation

Table 5 reports percentage of those who answered yes or no for each income category. It shows that labour income is the most important source of income for older adults, while non-labour income (i.e., pension, insurance and lottery income) and assets income account for only a small fraction of their total income. For example, over 55% of people aged 50+ reported receiving labour income in 2000 and this proportion increased to almost 62% in 2014. In contrast, only 6 to 7% of older adults had assets income in 2000 and while those numbers increased slightly in 2014, they were still less than 10% (9.93% for people aged 50+ in 2014). This fact is consistent with the observation that a large proportion of older adults are still working at older age. It also shows a changing composition of income sources by age group, with younger cohorts relying even more on labour income while more older cohorts receiving positive net transfers from them children.

Similar findings can be observed from Table 6, which shows percentage of each income source in total household income. These results directly relate to Fig. 6 in the main text (here also reported for IFLS wave 2007).

## Footnotes

1 There are a variety of approaches to poverty measurement, and these generate different outcomes. For example, based on an alternative “basic needs” poverty line measure developed by Allen (2017), Indonesia’s overall poverty rate is very high, at 46%.

2 Kudrna et al. (forthcoming) provide an up-to-date account of pension policy developments in East and South East Asia.

3 Data can be downloaded at https://population.un.org/wpp/Download/Standard/Population/.

4 Data and documentations can be accessed at https://www.rand.org/well-being/social-and-behavioral-policy/data/FLS/IFLS.html.

5 Note that the IFLS covers the whole life cycle, collecting data for household heads aged 18 and over, whereas the Health and Retirement Survey (HRS) in the US and many other HRS-related surveys in other countries cover only older ages - 45 and over.

6 In this paper, we employ mainly the last three waves of the IFLS (2000–2007-2014), which provide the most updated information and necessary background for the analysis of both labour force and older adults in Indonesia. In comparison with the earlier waves (1993–1997), those waves have more specific questions in relation to constructing the respondents’ sectoral attainment (formal/informal labour), which is in accordance with the definition by the ILO. However, note that for certain specific issues, we also report the 1993–1997 waves in order to capture specific trends and supporting information.

7 It is important to note that informal employment during the working years has important implications for older adults, particularly for their employment and sources of income at older ages.

8 For further details on measuring informality, see, e.g., ILO (2013; 2018a).

9 Most of the analysis related to the Indonesian labour force (Section 3.2) deals with male workers only (with the reasons discussed in that section). In Section 4 on older adults and the appendix, we provide a comparison by gender.

10 The (alternative) legal status of the firm definition of informality has been used, for example, by Gultom (2014) and Rothenberg et al. (2016), documenting a large and persistent informal sector in Indonesia, using firm-level data. Rothenberg et al. account for different-size firms (based on a number of employees) and show that the share of informal firms increases for small and micro firms (with 5–19 employees and less than 5 employees, respectively) to over 93% and 96%, respectively.

11 Note that according to ILO (2018b) (drawing on the analysis with SAKERNAS), the proportion of Indonesians in informal employment operating in the formal sector (based on the registered enterprise definition given above) is small, 5.8% of all informal employment. Under the recent social security reforms implemented in 2014 and 2015, the objective is to gradually cover all formal sector workers by the national social security programs, including health, work-accident, old-age savings, pension and death benefits programs (for details, see Muliati & Wiener, 2014).

12 Source: https://www.rand.org/well-being/social-and-behavioral-policy/data/bps/sakernas.html.

13 Source: https://www.rand.org/well-being/social-and-behavioral-policy/data/bps/susenas.html.

14 As a follow-up project, we will also use SAKERNAS and SUSENAS to complement the analysis of older adults carried out with the IFLS, and to establish the model-based structure of the labour market, sectoral transitions, skills and labour supply, earnings of the working age population and family transfers between generations (for the labour market and pension policy analysis applied to Indonesia).

15 In this paper, we employ the UN data since this data source is publicly available and allows for international comparison. There are also official projections for Indonesian population produced by BPS-Statistics Indonesia and Bappenas. Note that the latest population projection for Indonesia was produced for the period of 2015–2045 and released in 2018. This official projection is based on population census in 2010 and the 2015 Indonesian population survey between censuses (SUPAS). For comparison of the UN population projections and the population projections based on population census data in Indonesia, see McDonald (2014).

16 Projected rates (for 2020–2100) are based on the UN medium (most likely) population projection.

17 This is driven by substantial changes in age-specific fertility rates since 1960–65. Specifically, there was a substantial drop in the fertility rates among women aged 15–34, with a projected shift of peak fertility toward the age group 30–34 UN, 2019.

18 Similar increases can be shown for other life expectancy measures such as life expectancy at birth. In Indonesia, the life expectancy at birth is projected to reach almost 80 years (average for both sexes) in 2100 (UN, 2019). The difference here is that the life expectancy at birth has increased in the last century, particularly due to falling mortality rates at birth and very young ages. However, the projected improvements in survival rates at older ages will drive exponential increases in life expectancy of older adults.

19 Note that the inverse of the age dependency ratio is the potential support ratio that indicates how many people of working age there are to “potentially” support the elderly.

20 Currently about 12% of the total Chinese population is aged 65+, but the elderly population share is expected to reach almost 32% in 2100 (UN, 2019). The increases in elderly population shares that are projected for Indonesia and Vietnam are even more pronounced, e.g. in Vietnam, increasing from around 8% now to almost 30% in 2100.

21 In Indonesia, the total population size has almost quadrupled, increasing to 273 million people in 2020, from 69.5 million people in 1950. Under the base case (or the medium (most likely) scenario) projection, its total population is projected to further increase to 325 million people in 2100, but the annual population growth rate will become negative, with the total population declining at the annual rate of about −0.3% projected for 2100. Under the low-fertility projection for 2100, the total population will decline to about 200 million and the annual population growth rate will be −1.2% (in 2100). Further details on UN demographic projections for Indonesian population structure, size and growth (using alternative demographic assumptions) were relegated to the Appendix.

22 Specifically, in ILO (2018a, b), all own-account employers and all contributing family members are classified as in informal employment, and for employees to be considered as informal, the employment relationship should not be subject to national labour legislation, income taxation, social contributions and/or entitlement to certain employment benefits (see ILO (2018b) p.9). Note that we use more relaxed definition for informal “employees” based on only entitlement to medical benefits from their employers.

23 In Kudrna et al. (2020), we also provide data for females, showing that their employment rate (over working ages) has increased significantly over the last two decades.

24 Note that the retirement age (normal pension age) was increased to 56 in 2012 for employees in the private sector. Currently, the retirement age is 57 years and it has been legislated to gradually increase to age 65 by 2043 (OECD, 2018). For public servants, the standard retirement age was 56, but they could retire at age 50, with a minimum of 20 years of service (Muliati & Wiener, 2014).

25 This definition differs to those commonly used in OECD countries where those defined as high skill have some tertiary education (OECD, 2020). Further note that according to World Bank (2020a), median years of schooling for Indonesian males aged 25+ equaled to less than ten in 2012. We use 12 years of schooling as a cut-off. Note that this is now a mandated minimum school attendance in Indonesia.

26 Recall that for employees, we used only medical benefits and/or health insurance from employers as the criterium determining formal vs. informal employment. It should be pointed out that adding formal pension policy coverages (to our criterium) would further increases the proportion of informal workers. Note that in 2014, Indonesia introduced a pension policy reform extending pensions to all formal sector workers, which is now being gradually phased in. Hence, some of those reported in this paper as formal would not be subject to formal (tax and) pension policy during the period of the IFLS. Nevertheless, our figures for the size of informality in Indonesia are not that different from other studies, using different data sets. For example, the Asian Development Bank (2011) reported that 75.90% and 89.14% of the total employment (jobs) were informal in the Indonesian provinces of Banten and Yogyakarta, respectively, using Sakernas and the Informal Sector Survey (ISS) data. More recently, ILO (2018b) (with broader definition of informal employees) reported that 85.6% of Indonesians operate in informal employment, with 67.5% operating in the informal sector, 5.8% in the formal sector (based on the registered enterprise definition) and 12.2% in households (producing goods exclusively for own final use by their households).

27 When differentiating workers by age, we see that informality has a u-shaped profile, higher at very young ages, lower at middle age but increasing significantly at older ages. At older ages, this is partly due to pension policy with low formal sector retirement age (at age 55 during the period of the ILFS coverage). As for the skill distribution by age, older cohorts are less likely to be classified as high skill, with the proportion of high skill shown to decline with age. These cohort-specific results can be requested from the authors.

28 That’s despite the economic growth in Indonesia averaging over 5% p.a during that period (World Bank, 2020a).

29 In Kudrna et al. (2020), we have assumed somewhat different data selection and constructed a sample of men aged 25 to 60 (older cohorts). The overall share of informal employment averages around 81% (compared to about 77.4% here). The decline in informal employment over the period of 2000 to 2014 is about the same in percentage terms. Hence, the assumption of older cohorts aged 25–60 (compared to 20–54) increases informality, with almost all workers aged 55–60 being informal, but it also increases the share of low skill workers both in formal and informal employment ..

30 The labour earnings are reported in Indonesia Rupiah (IDR) p.a., but we take natural log (ln) and work with that measure.

31 The data is available at: https://data.imf.org/?sk=4FFB52B2-3653-409A-B471-D47B46D904B5

32 See page 1 (Preamble) of ILO Recommendation R204 (2015), available at: https://www.ilo.org/dyn/normlex/en/f?p=NORMLEXPUB:12100:0::NO::P12100_ILO_CODE:R204

33 This, to a large extent, supports the duel economy theory according to which formal and informal firms are largely different, producing different goods with different inputs and servicing different customers (see La Porta and Shleifer (2014), with empirical evidence of this model provided for Indonesia by Rothenberg et al., 2016).

34 According to Frankenberg et al. (2002), in a survey of experts in “adat” (traditional law) conducted in some 250 communities as part of the IFLS, 99% of respondents reported that traditional law required children to care for older parents.

35 As mentioned, more detailed results for employment of older adults are provided in Appendix, also showing employment rates of different cohorts and different type of employment (part-time vs full time), which also include results from other IFLS waves (than those in Figure 5).

36 Similar findings of a large gap between employment rates of men and women in Indonesia have been also reported in Australia Indonesia Partnership for Economic Governance (AIPEG) (2017p. 8), with their analysis based on SUSENAS 2013, and ILO (2017).

37 Assuming the annual time endowment of 5460 h (15 non-sleeping hours per day), males aged 60–64 on average supplies almost 37% of their annual time to labour and for those older aged 65–74 this is around 30%. This is much higher than observed labour supply of at those ages in developed countries (OECD, 2020).

38 In the analysis, we have also removed (i) all those with negative total income and (ii) 1% at the bottom of the total income distribution and 1% at the top of total income distribution.

39 For further details and discussion on income sources at older age see the Appendix, also including Table 5 that reports whether (or not) older households at different age groups (in different survey years) receive income from labour, non-labour (defined above) and assets and net transfers from their children.

40 There is a large literature studying intergenerational family transfers and the motives for family transfers (i.e. pure altruism, impure altruism with exchange motive, and explicit reciprocity) in the context of developing countries. For example, Lillard and Willis (1997) find strong evidence of the parental repayment hypothesis in Malaysia, showing that both the probability and the amount of transfers to elderly parents increase with higher educational attainment of their adult children. The consistent exchange motive has been noted by Secondi (1997) for China and Hoddinott (1992) for Kenya, while Cai et al. (2005) find evidence of an altruistic motive for private transfers at very low level of household income in urban China. Ravallion and Dearden (1988) show that the inter-generational transfers on the Indonesian island of Java are generally aimed at individuals suffering from an illness. Other motives for transfers such as pure loan contract and self-enforcing reciprocity are studied by Raut and Tran (2005), using the IFLS. Also using the IFLS data, Cameron and Cobb-Clark (2008) examines the labour supply of elderly Indonesians and find little evidence on the relation between the reduction of parents’ labour supply and support from their children – either through co-residence or financial transfers. Using Vietnamese data, Nguyen et al. (2012) show that transfers from children help older adults cope with risks related to old-age and illness.

41 See page 1 (Preamble) of ILO Recommendation R204 (2015), available at: https://www.ilo.org/dyn/normlex/en/f?p=NORMLEXPUB:12100:0::NO::P12100_ILO_CODE:R204

42 This, to a large extent, supports the duel economy theory according to which formal and informal firms are largely different, producing different goods with different inputs and servicing different customers (see La Porta and Shleifer (2014), with empirical evidence of this model provided for Indonesia by Rothenberg et al., 2016).
